# Transiently Observed Trace Albuminuria on Urine Dipstick Test Is Associated With All-Cause Death, Cardiovascular Death, and Incident Chronic Kidney Disease: A National Health Insurance Service-National Sample Cohort in Korea

**DOI:** 10.3389/fcvm.2022.882599

**Published:** 2022-05-02

**Authors:** Samel Park, Jiyoung Woo, Subeen Leem, Nam Hun Heo, Nam-Jun Cho, Hyowook Gil, Jae Heon Kim, Eun Young Lee

**Affiliations:** ^1^Department of Internal Medicine, Soonchunhyang University Cheonan Hospital, Cheonan, South Korea; ^2^Department of Bigdata Engineering, Soonchunhyang University, Asan, South Korea; ^3^Department of Biostatistics, Soonchunhyang University Cheonan Hospital, Cheonan, South Korea; ^4^Department of Urology, Soonchunhyang University Seoul Hospital, Seoul, South Korea; ^5^Institute of Tissue Regeneration, College of Medicine, Soonchunhyang University, Cheonan, South Korea; ^6^BK21 Four Project, College of Medicine, Soonchunhyang University, Cheonan, South Korea

**Keywords:** albuminuria, low-grade albuminuria, cardiovascular mortality, all-cause mortality, chronic kidney disease

## Abstract

**Introduction:**

Albuminuria is a well-known risk factor for end-stage kidney disease, all-cause mortality, and cardiovascular mortality, even when the albumin-to-creatinine ratio is <30 mg/g. However, the association between transiently observed trace albuminuria and these major adverse outcomes has not yet been reported. This study aimed to examine the effect of transient albuminuria on these major adverse outcomes using the National Health Insurance Service data in Korea.

**Methods and Results:**

The National Health Insurance Service-National Sample Cohort from Korea, followed from 2002 to 2015, consisted of 1,025,340 individuals, accounting for 2.2% of the total Korean population. We analyzed the effect of transient albuminuria on all-cause death, cardiovascular death, and incident chronic kidney disease (CKD) and compared it with the group without albuminuria. Among 1,025,340 individuals, 121,876 and 2,815 had transient albuminuria and no albuminuria, respectively. Adjusted hazard ratios of the transient albuminuria group for cardiovascular death and incident CKD were 1.76 (1.01–3.08) and 1.28 (1.15–1.43), respectively. There were significant differences in all-cause death, cardiovascular death, and incident CKD between the two groups after propensity score matching (*p* = 0.0037, *p* = 0.015, and *p* < 0.0001, respectively). Propensity score matching with bootstrapping showed that the hazard ratios of the transient albuminuria group for all-cause death and cardiovascular death were 1.39 (1.01–1.92) and 2.18 (1.08–5.98), respectively.

**Conclusions:**

In this nationwide, large-scale, retrospective cohort study, transient albuminuria was associated with all-cause death, cardiovascular death, and incident CKD, suggesting that transient albuminuria could be a risk marker for adverse outcomes in the future, and that its own subclinical phenotype could play an important role during the course of CKD.

## Introduction

Albuminuria is a well-known risk factor for end-stage kidney disease (ESKD), all-cause mortality, and cardiovascular mortality (1–6). It is a surrogate marker for predicting a decline in the glomerular filtration rate (GFR); if albuminuria is managed inadequately, it will continue to increase, culminating in a concomitant decrease in GFR (7–9). Since albuminuria is an indirect marker of glomerular filtration barriers, its presence corroborates impairments in glomerular filtration barriers, including endothelial cells and their glycocalyx system, the glomerular basement membrane, and podocytes, and implies problems in interactions among them (10).

It has been shown that not only macroalbuminuria (albumin excretion rate, AER > 300 mg/day or albumin-to-creatinine ratio, ACR > 300 mg/g) and microalbuminuria (AER 30–300 mg/day or ACR 30–300 mg/g), but also an ACR of 10–30 mg/g are associated with mortality and ESKD (3, 5). As such, albuminuria, defined as ACR <30 mg/g, once indicated low-grade albuminuria (11). The Kidney Disease: Improving Global Outcomes chronic kidney disease (CKD) workgroup has recommended a CKD classification based on albuminuria and GFR (12). They emphasized the detrimental effects of albuminuria; thus, the guideline recommends using the terms “moderately increased” and “severely increased” instead of microalbuminuria and macroalbuminuria. In addition, normoalbuminuria (or low-grade albuminuria) was replaced by “normal to mildly increased” (12).

Transient albuminuria can be observed in disorders other than CKD, including symptomatic urinary tract infection, exercise, fever, seizure, heart failure, diurnal variation, and other conditions that can increase vascular permeability (12, 13). Since albuminuria is a marker of glomerular hyperfiltration, which implies the initiation of a kidney injury (14), and is attributed to leakage through a damaged area of the glomerular filtration barrier, transient albuminuria might also be caused by injury to the glomerular barrier of the kidney. Therefore, an association between transient albuminuria and cardiovascular mortality, and even all-cause mortality, can be inferred.

Studies on the effect of transient albuminuria on major adverse outcomes are scarce. A recent report showed that transient dipstick proteinuria affects cardiovascular mortality, but not all-cause mortality (15). In that study, participants who initially noticed a result of ≥1+, which then decreased to negative or trace in the urine dipstick test, were defined as the transient dipstick proteinuria group. A previous study reported that trace albuminuria in the urine dipstick test is associated with mortality (3). Data from Korea reveal that low-risk individuals with trace urine in the dipstick test had a hazard ratio (HR) of all-cause mortality that was ~20% higher than those with negative urine (16). Given that trace albuminuria in the urine dipstick test was associated with mortality, separating proteinuria by a dipstick result of ≥1+ or negative/trace was inappropriate. Therefore, we examined the effect of transiently observed trace albuminuria in the urine dipstick test on major adverse outcomes using the National Health Service (national insurance) data in Korea.

## Materials and Methods

The study protocol was reviewed and approved by the Institutional Review Board (IRB) of Soonchunhyang University Cheonan Hospital (Cheonan, Korea) (IRB No: SCHCA 2019-04-030) and the need for informed consent was waived by IRB because of the retrospective study design. The study was conducted in accordance with the principles of the Declaration of Helsinki. Data were accessed *via* the National Health Insurance Sharing Service after an adequate approval process.

### Study Population

We analyzed data from the National Health Insurance Service-National Sample Cohort (NHIS-NSC) in Korea (17). The NHIS-NSC is a sampled cohort of 1,025,340 individuals based on the National Health Information Database and is composed of general health examination data of the individuals; the prescription lists, except over-the-counter drugs; and the International Classification of Diseases 10th revision (ICD-10) codes (17, 18). The cohort was followed up for 13 years, from 2002 to 2015. However, since data on serum creatinine levels is available only since 2009, we targeted the population since 2009, including those individuals aged >20 years. As the data in this cohort were unstructured, a detailed description of the study population, data construction, collected variables, and analyses have been included in the Supplementary Materials.

Results of the urine dipstick test showing negative (–), trace (±), positive (1+), and positive (≥2+) were considered to correspond to ACR levels of <10, 10–29, 30–299 (microalbuminuria), and ≥300 mg/g (macroalbuminuria), respectively (19). Since individual-level health examination programs are performed biennially, we combined 2 consecutive years into one observation unit for the years 2005–2006, 2007–2008, and 2009–2010. When patients had negative urine dipstick test results (i.e., –/–/–) during three observational periods, they were assigned to the no albuminuria group. If patients had trace (±) results in one of the first two observational periods and were negative in others (i.e., ±/–/– or –/±/–), they were assigned to the transient albuminuria group. If the urine dipstick test was negative in both the first two sequential observational periods, but showed a trace result in the last observational period (i.e., –/–/±), the patients were excluded from the analysis because the results of the follow-up urine dipstick tests were uncertain.

### Statistical Analyses

The third observation period (2009–2010) was selected as the baseline. The outcomes of interest were as follows: (1) all-cause death, (2) cardiovascular death, (3) incident CKD, and (4) decline in estimated GFR (eGFR) by more than 30%. All-cause death was defined as death due to any cause, excluding those with an S or T code, according to the ICD-10 codes. Cardiovascular death was defined as death with the leading cause recorded as I code by ICD-10 (I00–I99). Incident CKD was defined as either eGFR or albuminuria. The age-adapted eGFR threshold was used as the eGFR criteria (20). Based on previous studies, albuminuria was defined as a urine dipstick test result of ≥1+ (21, 22). A decline in eGFR by ≥30% was also based on previous studies, although the strength of evidence was slightly weaker than that of doubling of serum creatinine or a decline in GFR by ≥40% (23).

The stratified Cox proportional analysis was used. The no albuminuria group was used as a reference for comparison with the transient albuminuria group. Among the baseline characteristics, age, sex, residence (urban vs. rural area), income level estimated based on health insurance expenditures (deciles), BMI (kg/m^2^), systolic blood pressure (mmHg), eGFR calculated using the Chronic Kidney Disease Epidemiology Collaboration equation (24), the Charlson comorbidity index (CCI) (25), hypertension (HTN), diabetes mellitus (DM), alcohol consumption, and smoking history were used as confounding factors.

All statistical analyses were performed using R version 3.3 (The R Foundation for Statistical Computing, Vienna, Austria). Categorical variables were expressed as counts and percentages. Continuous variables were expressed as mean ± standard deviation (SD). The differences between groups were compared using the Student's *t*-test or Mann-Whitney test, as appropriate. We used 1:3 propensity matching and the nearest method with a caliper of 0.25 (26). The propensity score was regressed with baseline characteristics considered as covariates to be adjusted for in the Cox proportional hazard model because covariates affecting outcomes are better for estimating the propensity score to regress the treatment group (27–29). Matching between the two groups was based on propensity scores using the “MatchIt” package of the R software. Survival analysis was performed using the stratified log-rank test based on propensity scores (30). When the difference in the number of participants between the two groups was large, there was a concern that the result might vary depending on which individuals were selected during the matching process. To address this problem, a sensitivity analysis using bootstrapping was performed. Our simulation process was adopted from a previous study (31). The mean HR and 95% confidence intervals (CIs) were obtained by non-parametric percentile-based estimates (i.e., 2.5th and 97.5th percentiles) through a combination of propensity score matching and bootstrapping.

## Results

### Study Population

**Figure 1:**
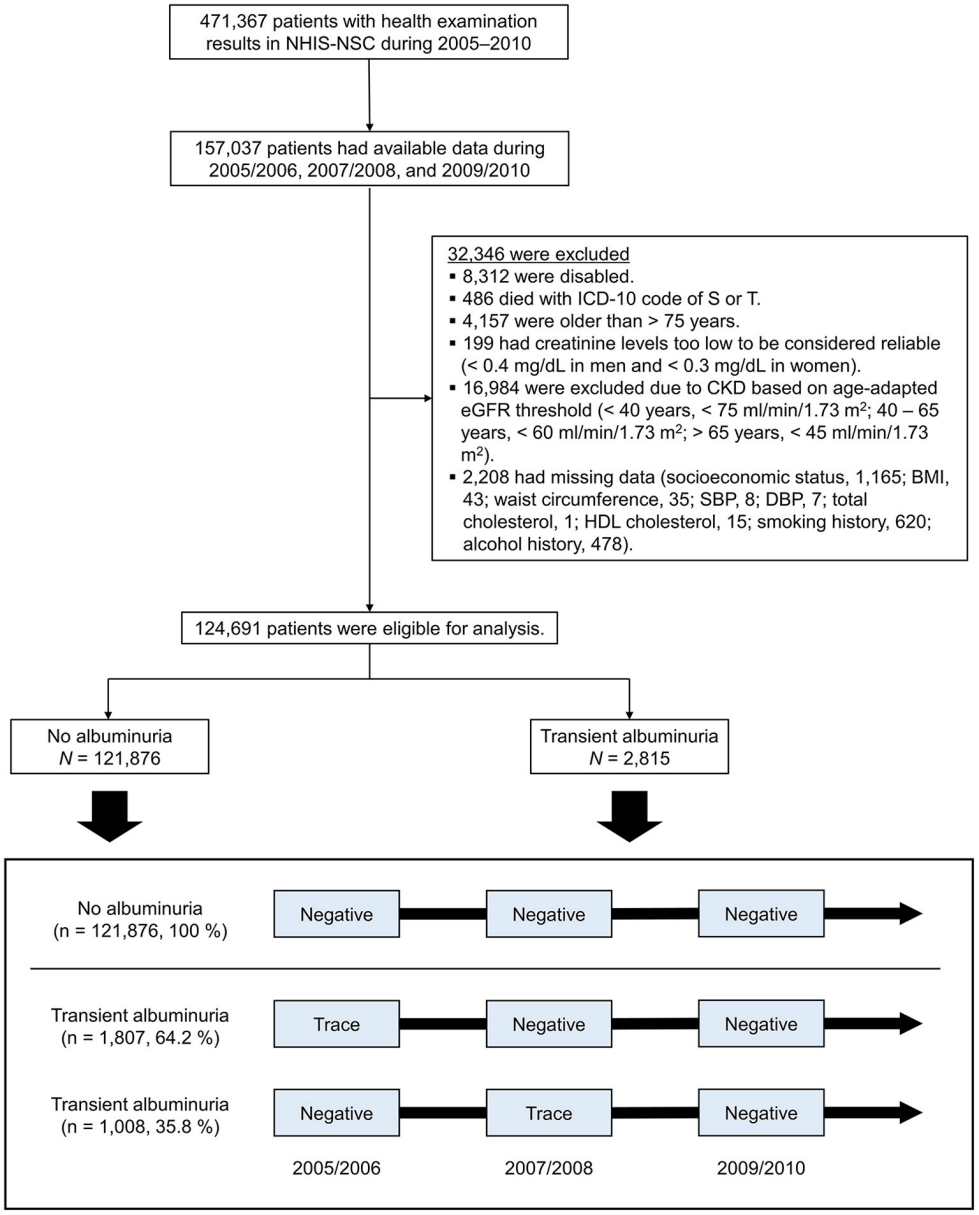
Flow chart depicting a diagram of patient enrollment. The box shows how the observation period was structured. NHIS-NSC, National Health Insurance Service-National Sample Cohort; ICD-10, International Classification of Diseases, 10th revision; CKD, chronic kidney disease; eGFR, estimated glomerular filtration rate; BMI, body mass index; SBP, systolic blood pressure; DBP, diastolic blood pressure; HDL, high-density lipoprotein.

We screened 1,025,340 individuals, and only 124,691 were eligible for this study. Figure 1 depicts the patient selection process. Among those included in the study, 121,876 people were in the no albuminuria group (–/–/– during the observation period) and 2,815 were in the transient albuminuria group (1,807 with ±/–/– and 1,008 with –/±/– during the observation period). During the follow-up period, the serum creatinine levels and the urine dipstick test results of the subjects were measured two times (IQR: 3–4).

The baseline clinical characteristics are presented in Table 1. Age, weight, BMI, waist circumference, and systolic blood pressure were higher, and eGFR was lower in the transient albuminuria group than in the no albuminuria group. The transient albuminuria group also had a higher prevalence of HTN, DM, and severe comorbidity (as expressed by a higher CCI score) than the no albuminuria group. Detailed comorbidities (based on CCI score) of the patients are presented in Supplementary Table 1. Since the socioeconomic status inevitably affects participation in national health check-up programs, more individuals with higher income received national health screening services (Supplementary Table 2).

**Table 1:** Baseline characteristics of participants in the no albuminuria and transient albuminuria groups.

**Variable**	**No albuminuria**	**Transient albuminuria**	***p*-value**
Participants, *n*	121,876	2,815	
**Clinical and demographic characteristics**			
Mean age (SD), year	48.4 (12.4)	49.2 (12.3)	0.001
**Age group**, ***n*** **(%)**			
20–39 yr	32,949 (27.0)	712 (25.3)	0.099
40–59 yr	62,843 (51.6)	1,473 (52.3)	
60–75 yr	26,084 (21.4)	630 (22.4)	
Male, *n* (%)	69,938 (57.4)	1,566 (55.6)	0.066
Residence, urban area, *n* (%)	79,437 (65.2)	2,125 (75.5)	<0.001
Mean height (SD), cm	164.2 (9.1)	164.0 (9.0)	0.244
Mean weight (SD), cm	64.3 (11.3)	65.3 (11.9)	<0.001
Mean body mass index (SD), kg/m^2^	23.7 (3.1)	24.2 (3.3)	<0.001
<18.5, *n* (%)	3,678 (3.0)	84 (3.0)	<0.001
18.5–24.9, *n* (%)	78,307 (64.3)	1,653 (58.7)	
25–29.9, *n* (%)	36,326 (29.8)	948 (33.7)	
≥30, *n* (%)	3,565 (2.9)	130 (4.6)	
Mean waist circumference (SD), cm	80.5 (8.7)	81.6 (9.4)	<0.001
Mean systolic BP (SD), mmHg	122.2 (14.5)	122.8 (15.0)	0.026
Mean diastolic BP (SD), mmHg	76.4 (9.8)	76.7 (10.3)	0.087
Mean arterial BP (SD), 10 mmHg	91.6 (10.7)	92.1 (11.2)	0.048
Mean creatinine level (SD), mg/dL	0.9 (0.2)	0.9 (0.2)	0.286
Mean eGFR (SD), mL/min per 1.73 m^2^	90.0 (15.7)	89.0 (16.1)	0.001
45–59, *n* (%)	1,795 (1.5)	54 (1.9)	<0.001
60–74, *n* (%)	19,875 (16.3)	538 (19.1)	
75–89, *n* (%)	41,328 (33.9)	955 (33.9)	
90–104, *n* (%)	36,732 (30.1)	755 (26.8)	
≥105, *n* (%)	22,146 (18.2)	513 (18.2)	
**Medical history**			
Hypertension, *n* (%)	22,420 (18.4)	700 (24.9)	<0.001
Diabetes, *n* (%)	8,491 (7.0)	286 (10.2)	<0.001
Current smoker, *n* (%)	29,521 (24.2)	640 (22.7)	0.072
Alcohol intake, *n* (%)			0.058
None	62,338 (51.1)	1,485 (52.8)	
Moderate	38,249 (31.4)	824 (29.3)	
Heavy	21,289 (17.5)	506 (18.0)	
The Charlson comorbidity index (SD), score	0.4 (0.8)	0.4 (0.9)	0.010

*Continuous variables are presented as mean (SD). Categorical variables are presented as number (%).*

*BP, blood pressure; eGFR, estimated glomerular filtration rate; SD, standard deviation*.

### Association With All-Cause Death and Cardiovascular Death

**Figure 2:**
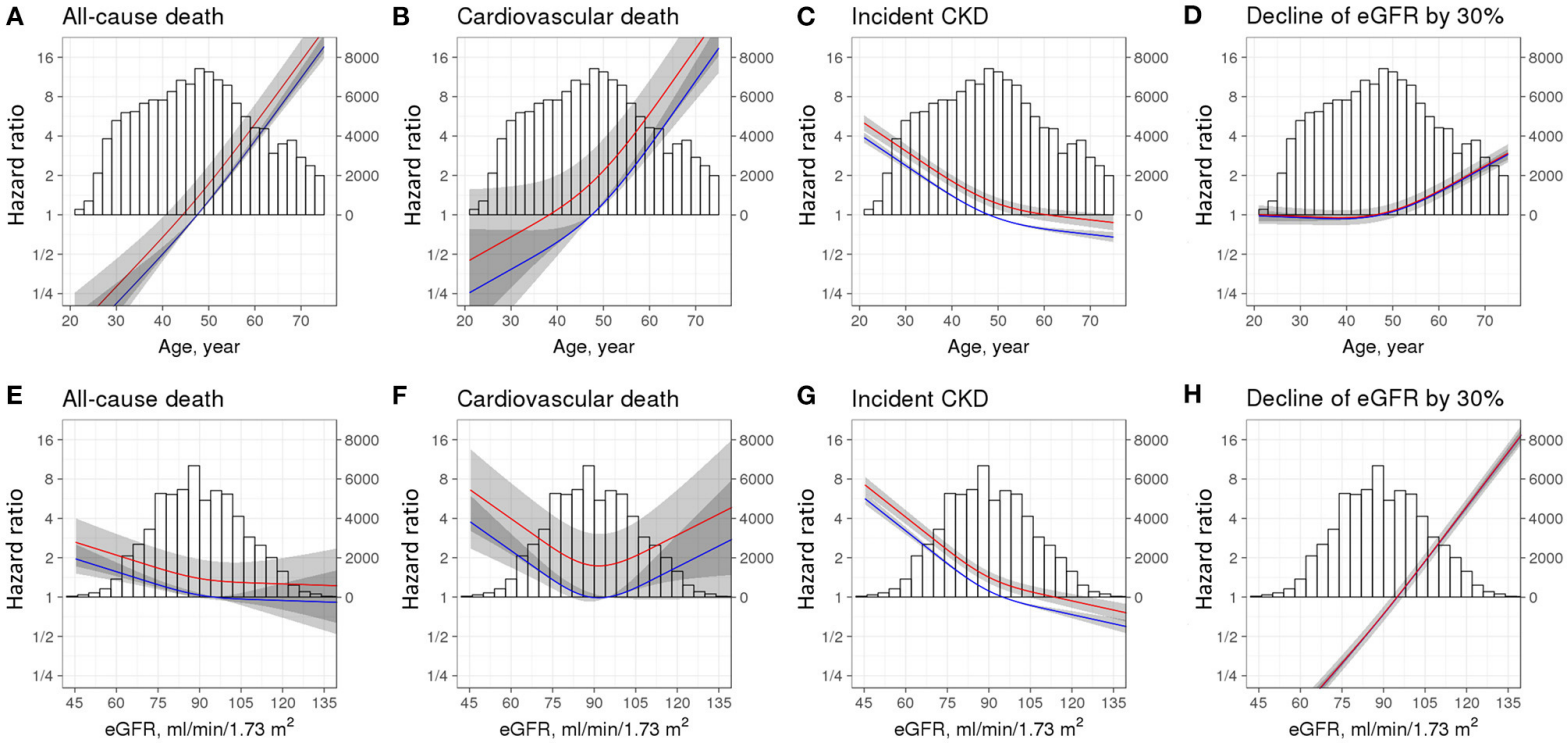
Restricted cubic spline curves for adverse outcomes according to age, estimated glomerular filtration rate (eGFR), and groups. Hazard ratios and 95% confidence intervals were drawn according to spline age **(A–D)** and eGFR **(E–H)**. Adverse outcomes included all-cause death **(A,E)**, cardiovascular death **(B,F)**, incident CKD **(C,G)**, and decline of eGFR by 30% **(D,H)**. Models were adjusted for age, sex, residence, income level, BMI, systolic BP, eGFR, Charlson comorbidity index score, history of HTN and DM, and smoking and alcohol status. The reference was age of 47.5 years **(A–D)** and eGFR of 95 ml/min per 1.73 m^2^ **(E–H)** plus no albuminuria group. Red line, transient albuminuria group; Blue line, no albuminuria group. CKD, chronic kidney disease; eGFR, estimated glomerular filtration rate; BMI, body mass index; BP, blood pressure; HTN, hypertension; DM, diabetes mellitus.

The incidence rates of all-cause death were 357 and 254 cases per 100,000 person-years in the transient and no albuminuria groups, respectively (Table 2). Cardiovascular death also developed more frequently in the transient albuminuria group (97 cases per 100,000 person-years) than in the no albuminuria group (49 cases per 100,000 person-years). In the unadjusted Cox proportional hazard models, transient albuminuria was associated with all-cause death [1.41 (1.06–1.88)] and cardiovascular death [1.97 (1.13–3.43)] (Table 2). However, in the adjusted model, only the association between transient albuminuria and cardiovascular death remained significant [1.76 (1.01–3.08), *p* = 0.048]. The results of the model in which the non-HDL cholesterol level was additionally adjusted as a covariate were comparable with the results the model without non-HDL cholesterol adjustment (172 individuals were excluded because their non-HDL cholesterol levels were ≤ 0) (Supplementary Table 3). The HRs for all-cause death (Figure 2A) and cardiovascular death (Figure 2B) increased with age. As the baseline eGFR decreased, the relative risk of all-cause death increased (Figure 2E). However, the HR between cardiovascular death and the baseline eGFR was U-shaped (Figure 2F).

**Table 2:** Relative hazard ratio of outcome in the transient albuminuria group compared to that in the no albuminuria group.

**Outcome**	**Number of events**	**Crude incidence rates**	**Hazard ratio^**†**^**	***p*-value**	**Hazard ratio^**‡**^**	***p*-value**
**All-cause death** ^**§**^						
No albuminuria	1,502 (1.2)	254 (241–267)	Reference		Reference	
Transient albuminuria	48 (1.7)	357 (256–457)*	1.41 (1.06–1.88)	0.020	1.34 (0.96–1.88)	0.090
**Cardiovascular death** ^**||**^						
No albuminuria	291 (0.2)	49 (44–55)	Reference		Reference	
Transient albuminuria	13 (0.5)	97 (44–150)	1.97 (1.13–3.43)	0.017	1.76 (1.01–3.08)	0.048
**Incident CKD** ^**#**^						
No albuminuria	11,835 (9.7)	2,111 (2,073–2,149)	Reference		Reference	
Transient albuminuria	363 (12.9)	2,892 (2,595–3,190)**	1.37 (1.23–1.52)	<0.001	1.28 (1.15–1.43)	<0.001
**>30% decline in eGFR** ^**††**^						
No albuminuria	10,344 (8.5)	1„825 (1789–1,860)	Reference		Reference	
Transient albuminuria	237 (8.4)	1,842 (1,607–2,076)	1.01 (0.89–1.15)	0.901	1.02 (0.86–1.12)	0.741

*Age, sex, residence, income level, BMI, systolic BP, baseline eGFR, Charlson comorbidity index score, previous history of HTN and DM, and smoking and alcohol status were included in the model to adjust for other confounders. Whether confounders would be used as covariates or strata was determined based on Schoenfeld residuals.*

*P < 0.05, **P < 0.001.

*^†^Hazard ratio for a crude model.*

*^‡^Hazard ratio for an adjusted model.*

*^§^BMI, systolic BP, and baseline eGFR were used as covariates and others were used as strata.*

*^||^Residence, BMI, systolic BP, baseline eGFR, smoking and alcohol status, previous history of HTN and DM, and Charlson comorbidity index score were used as covariates and others were used as strata.*

*^#^Residence, BMI, systolic BP, baseline eGFR, smoking and alcohol status, and previous history of DM were used as covariates and others were used as strata.*

*^††^Residence, BMI, systolic BP, previous history of DM, and Charlson comorbidity index score were used as covariates and others were used as strata.*

*CKD, chronic kidney disease; eGFR, estimated glomerular filtration rate; BMI, body mass index; BP, blood pressure; HTN, hypertension; DM, diabetes mellitus*.

A total of 8,442 participants from the no albuminuria group were selected to match the transient albuminuria group based on the propensity score (Table 3). The matching process between the two groups was performed well and the distribution of propensity scores between the two groups was similar (Supplementary Figures 1A,B). One individual was discarded from the transient albuminuria group (Supplementary Figure 1C). After propensity score matching, there were no significant differences in the baseline characteristics between the two groups, including age, sex, residence, income level, BMI, systolic blood pressure, baseline eGFR, CCI score, history of HTN and DM, and smoking and alcohol status (Table 3). In addition, all standardized differences were < 0.1, indicating the validity of the matching process (Supplementary Figure 1D). After matching, all-cause death and cardiovascular death were statistically significant in the stratified log-rank test based on propensity scores (*p* = 0.004 and *p* = 0.022, respectively, Supplementary Figures 2A,B).

**Table 3:** Baseline characteristics after propensity score matching between the transient albuminuria and no albuminuria groups.

**Variables**	**No albuminuria (*n* = 8,442)**	**Transient albuminuria (*n* = 2,814)**	***p*-value**
**Clinical and demographic characteristics**			
Mean age, (SD), year	49.1 (12.3)	49.2 (12.3)	0.783
Male, *n* (%)	4,711 (55.8)	1,566 (55.7)	0.904
Residence, urban area, *n* (%)	6,425 (76.1)	2,124 (75.5)	0.516
Mean height (SD), cm	163.9 (9.1)	164.0 (9.0)	0.738
Mean weight (SD), cm	65.1 (11.6)	65.3 (11.9)	0.781
Mean body mass index (SD), kg/m^2^	24.1 (3.1)	24.2 (3.3)	0.939
Mean waist circumference (SD), cm	81.2 (8.8)	81.6 (9.4)	0.069
Mean systolic BP (SD), mmHg	122.8 (14.7)	122.8 (15.0)	0.904
Mean diastolic BP (SD), mmHg	76.7 (9.9)	76.7 (10.3)	0.994
Mean arterial BP (SD), 10 mmHg	92.0 (10.8)	92.1 (11.2)	0.876
Mean creatinine level (SD), mg/dL	0.9 (0.2)	0.9 (0.2)	0.830
Mean eGFR (SD), mL/min per 1.73 m^2^	88.9 (15.5)	89.0 (16.1)	0.887
**Medical history**			
Hypertension, *n* (%)	2,076 (24.6)	699 (24.8)	0.810
Diabetes, *n* (%)	812 (9.6)	285 (10.1)	0.452
Current smoker, *n* (%)	1,941 (23.0)	640 (22.7)	0.806
**Alcohol intake**, ***n*** **(%)**			
None	4,479 (53.1)	1,484 (52.7)	0.856
Moderate	2,484 (29.4)	824 (29.3)	
Heavy	1,479 (17.5)	506 (18.0)	
Charlson comorbidity index (SD), score	0.4 (0.9)	0.4 (0.9)	0.517

*Continuous variables are presented as mean (SD). Categorical variables are presented as number (%).*

*BP, blood pressure; eGFR, estimated glomerular filtration rate*.

### Association With Renal Outcome

During the follow-up period, 12,198 patients developed incident CKD. Among them, 7,522 cases were defined by the age-adapted eGFR criteria, and 5,244 cases were defined as having albuminuria ≥1+. The incidence rate of overt albuminuria, defined as ≥1+ by the urine dipstick test, was 1,653 (95% CI: 1,431–1,875) cases per 100,000 person-years among participants with transient albuminuria and 871 (847–895) cases per 100,000 person-years among those with no albuminuria, and the difference was statistically significant (*p* <0.001). Newly developed CKD defined by the age-adapted eGFR criteria was similar between the two groups [1,370 (95% CI: 1,169–1,571) vs. 1,286 (95% CI: 1,257–1,316) in the transient albuminuria and no albuminuria groups, respectively].

The association between transient albuminuria and incident CKD was robust in the crude Cox proportional hazard model [HR: 1.37 (1.23–1.52), Table 2]. This association remained strong after adjusting for confounding factors [HR: 1.28 (1.15–1.43), Table 2]. However, the decline in eGFR by more than 30% was not significantly different between the groups. The addition of the non-HDL cholesterol level to the confounding factors did not alter the results [HR: 1.29 (1.16–1.43), Supplementary Table 3]. The results after propensity score matching were also comparable (Supplementary Figures 2C,D). The HR between incident CKD and age decreased as age increased (Figure 2C), while the relative risk of eGFR decline by 30% gradually increased over the age of 50 years (Figure 2D). Figure 2G shows an inverse correlation between incident CKD and the baseline eGFR; however, interestingly, the relative risk of decline of eGFR by 30% was higher in conditions with a higher baseline eGFR (Figure 2H).

### Sensitivity Analysis

**Figure 3:**
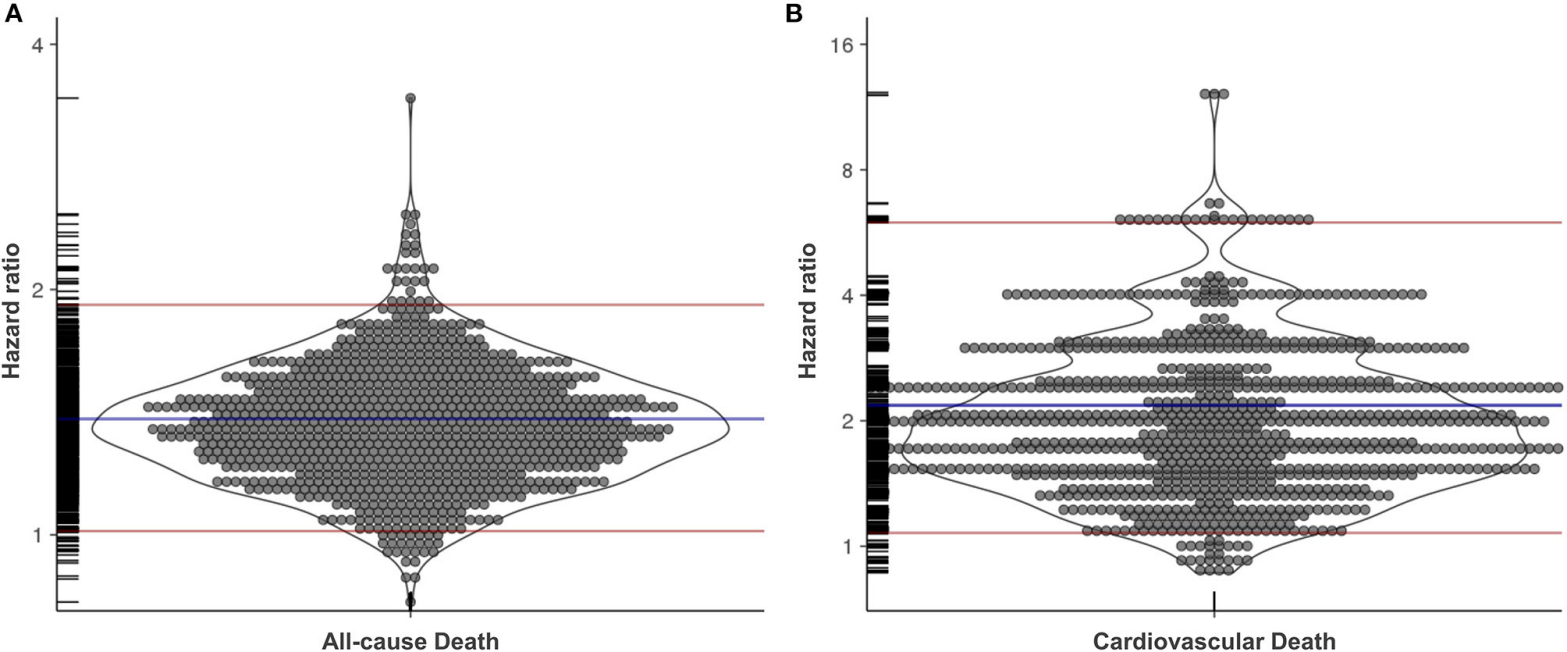
The hazard ratio of transient albuminuria on all-cause death and cardiovascular death after bootstrapping with propensity score matching. Each circle represents a hazard ratio for all-cause death **(A)** and cardiovascular death **(B)**. Blue lines and red lines represent mean and 95% confidence intervals, respectively. Rug plot for data is displayed along the y-axis.

Through simulation, 1,000 propensity score-matched sub-samples were created using bootstrapping. Each sub-sample consisted of ~15,000 participants. The HRs were calculated for each sub-sample and were 1.39 (1.01–1.92) and 2.18 (1.08–5.98) for all-cause death and cardiovascular death, respectively (Figure 3). Since no cardiovascular death occurred in the no albuminuria group in the two sub-sample sets, the coefficient beta converged to infinity. Thus, in these two cases, the HR could not be calculated. Consequently, a 95% CI was estimated using the remaining 998 HRs of each sub-sample. The results were similar after additional adjustment using non-HDL cholesterol, in which the HR for all-cause death was 1.39 (1.01–1.95) and that for cardiovascular death was 2.22 (1.01–6.07). The difference in outcome defined by incident CKD was notable between the two groups. However, there was no significant difference in the outcome defined by a 30% reduction in eGFR. Therefore, we did not perform a simulation using bootstrapping for these outcomes.

## Discussion

In this nationwide, large-scale, retrospective cohort study, transient albuminuria was associated with all-cause death, cardiovascular death, and incident CKD. The transient albuminuria group was significantly associated with cardiovascular death and incident CKD [HR: 1.76 (1.01–3.08) and 1.28 (1.15–1.43), respectively] after adjustment for confounding factors (Table 2). However, after propensity score matching, the transient albuminuria group was also significantly associated with all-cause death (*p* = 0.0037, Supplementary Figure 1A). The association between transient albuminuria and cardiovascular death and incident CKD remained after propensity score matching (*p* = 0.0215 and *p* < 0.0001, respectively, Supplementary Figures 1B,C). Since the inequality in sample sizes between the two groups could affect the results after propensity score matching, we used bootstrapping methods. The association between transient albuminuria and all-cause death [HR: 1.39 (1.01–1.92)] and cardiovascular death [HR: 2.18 (1.08–5.98)] were confirmed again after simulation using propensity score matching and bootstrapping. This is the first study to report a clinical association between transient albuminuria and other detrimental outcomes. A previous study showed that individuals with transient dipstick proteinuria, defined as those whose urine dipstick result increased from negative or trace to 1+ or greater, had an increased risk of cardiovascular mortality, but not all-cause mortality (15). However, since a trace urine dipstick test result is considered low-grade albuminuria to microalbuminuria (22), the previous study did not show a pure effect of transiently observed trace albuminuria.

Albuminuria, even at levels below 30 mg/g, is associated with all-cause mortality and cardiovascular mortality, even in non-hypertensive and non-diabetic individuals (11). Low-grade albuminuria has been shown to be directly associated with adverse cardiac mechanics, and especially with global longitudinal strain, E/e′ ratio (32), and left ventricular hypertrophy (33), indicating an association between low-grade albuminuria and cardiac remodeling (34). Additionally, low-grade albuminuria is associated with fatty liver (35). A nationwide population-based study in Korea showed that reducing components of metabolic syndrome could reduce the burden of CKD and cardiovascular events (36, 37), implying an association between low-grade albuminuria and metabolic components. Alternatively, a direct association between endothelial dysfunction and albuminuria may be involved (38). A loss of endothelial glycocalyx is associated with albuminuria and vascular endothelial cell dysfunction, linking albuminuria to adverse outcomes (39). In addition, albuminuria is associated with systemic inflammation. In patients with DM, systemic inflammation markers are increased preceding albuminuria (40). We previously reported that bariatric surgery can decrease albuminuria by reducing systemic inflammation in patients with severe obesity (41). Given previous evidence, the association between transient albuminuria and all-cause and cardiovascular death was convincing. As expected, all-cause death and cardiovascular death were more prevalent in older participants irrespective of the group (Figures 2A,B), and the inverse association between all-cause death and eGFR was reasonable. However, the association between cardiovascular death and eGFR was found to be U-shaped. A previous study also showed a U-shaped association between eGFR and all-cause death and between eGFR and cardiovascular death (3). This difference might be due to the use of the Chronic Kidney Disease Epidemiology Collaboration equation in our study.

Participants in the transient albuminuria group were associated with an increased risk of incident CKD, especially overt albuminuria, defined by ≥1+ in the urine dipstick test. However, renal outcomes defined using eGFR changes (by age-adapted eGFR threshold and a decline in eGFR by more than 30%) were similar between the groups (Table 2). The relative risk for a 30% decline in eGFR correlated positively with the baseline eGFR (Figure 2D), which was attributed to the fact that a small perturbation in creatinine levels could erroneously affect the creatinine-based eGFR, as discussed in our previous report (42). Albuminuria, defined as ≥1+ in the urine dipstick test, was considered significant in previous studies (21, 22), and the occurrence of ≥1+ in the urine dipstick test has been used as a renal outcome previously (43). Since albuminuria represents a functional impairment of the glomerular filtration barrier, the presence of transient albuminuria might implicate pressure overload on the glomerular filtration barrier that does not appropriately interact with the state of physiological perturbation of the glomeruli (10). Glomerular hyperfiltration increases shearing stress and induces podocyte detachment, culminating in renal injury with podocyte injury observed histologically as focal segmental glomerulosclerosis (44). Therefore, transient albuminuria might be a subclinical phenotype of CKD in a context similar to that of subclinical acute kidney injury (45). In line with this notion, previous studies have reported that a slight increase in albuminuria is associated with CKD or a more rapid decline in eGFR (46, 47). Although we could not investigate whether trace albuminuria in a urine dipstick test represented increased albuminuria by ACR, transient albuminuria could be a substantial implication of transient impairment of the glomerular filtration barrier.

This study had several strengths. First, we used a preconstructed cohort warranted for study fields, including sociology, economics, public health, and medicine (17). The cohort included almost one million participants from the South Korean population using systematically stratified random sampling and was based on nationwide health insurance data with longitudinal data to overcome the limitations of cross-sectional data. Second, we performed propensity score matching to adjust for confounding factors that might differ greatly between groups (28). Since the difference in the number of participants between the groups was large, we minimized the effect of random sampling on results using bootstrapping followed by propensity score matching (31). Third, a sufficiently long observation period, which could not be achieved with randomized controlled trials, was used. In particular, the analysis was conducted after a clear separation between the transient albuminuria and no albuminuria groups based on several urine tests over several years. This process is difficult to perform in randomized controlled trials. The association between adverse outcomes and these types of exposures can only be acquired in observational settings.

Our study had some limitations. First, it was based on retrospectively acquired data. Therefore, we were unable to exclude any bias. However, having a long observation period causes difficulty in performing randomized controlled trials. We adjusted for confounding factors as much as possible by using propensity score matching. However, this method is powerless against unknown or unmeasured confounding factors (48). To overcome this, we extracted a subgroup using bootstrapping and then performed propensity score matching to estimate the HR. Second, we could not calculate the effects of drugs due to excessive calculation intensity. Third, albuminuria was measured using a urine dipstick test rather than by ACR. Given the low sensitivity of the urine dipstick test, patients with moderately increased albuminuria might not be detected (21, 22). Although a low awareness due to the low sensitivity of the dipstick test might influence our results (49), several dipstick tests (at least three times or more) during a long-baseline observation period made our results robust. Fourth, all the laboratory measurements were not centralized. The samples were collected and measured by each local clinic or laboratory facility, but only the laboratory results were collected by the NHIS. Therefore, there was no information on how to collect samples and perform laboratory analysis. However, Korea is a developed country and their quality control in laboratories is reliable. Fifth, the important confounding factors, the history of cardiovascular diseases, such as heart failure, atrial fibrillation, and acute coronary syndrome (50–52), were only based on the CCI. The NHIS cohort consisted of the results of health examination and the administrative code (i.e., ICD-10) that was claimed during real practice. Thus, the specific disease state was not available. We could not help but use the administrative code to explore the medical history associated with cardiovascular diseases. Sixth, because only Koreans were included in this study, our results cannot be applied to other races. Further research is needed to estimate whether our results are reproducible in other races.

In conclusion, in this nationwide, large-scale, retrospective cohort study, transient albuminuria, defined as traces in the urine dipstick test, was associated with all-cause death, cardiovascular death, and incident CKD. Our results suggest that transient albuminuria could be a risk marker for future adverse outcomes. Furthermore, the subclinical phenotype could play an important role during the course of CKD.

## Data Availability Statement

Publicly available datasets were analyzed in this study. This data can be found at: https://nhiss.nhis.or.kr/bd/ab/bdaba021eng.do.

## Ethics Statement

The study involving human participants were reviewed and approved by Institutional Review Board (IRB) of Soonchunhyang University Cheonan Hospital (Cheonan, Korea) (IRB No: SCHCA 2019-04-030). Written informed consent for participation was not required for this study in accordance with the national legislation and the institutional requirements.

## Author Contributions

SP and EYL conceived the study. SP, JYW, and SBL performed the analysis. SP drafted the manuscript. NHH, NJC, and JHK advised on statistics. HWG and EYL oversaw the study. All authors reviewed and approved the drafted manuscript.

## Funding

This work was supported by a National Research Foundation (NRF) of Korea Grant funded by the Korean Government (Ministry of Science and ICT) (2020R1A2C2003438, 2019M3E5D1A02069071, and 2021M3E5D1A02015171) and the Soonchunhyang University Research Fund.

## Conflict of Interest

The authors declare that the research was conducted in the absence of any commercial or financial relationships that could be construed as a potential conflict of interest.

## Publisher's Note

All claims expressed in this article are solely those of the authors and do not necessarily represent those of their affiliated organizations, or those of the publisher, the editors and the reviewers. Any product that may be evaluated in this article, or claim that may be made by its manufacturer, is not guaranteed or endorsed by the publisher.

## Abbreviations

CKD: chronic kidney disease
ESKD: end-stage kidney disease
GFR: glomerular filtration rate
AER: albumin excretion rate
ACR: albumin-to-creatinine ratio
NHIS-NSC: National Health Insurance Service-National Sample Cohort
ICD-10: International Classification of Diseases 10th revision
eGFR: estimated GFR
CCI: the Charlson comorbidity index
SD: standard deviation
HR: hazard ratio
CI: confidence interval
BMI: body mass index
HDL: high-density lipoprotein.
