# Supplementary figures and images for: Transiently Observed Trace Albuminuria on Urine Dipstick Test Is Associated With All-Cause Death, Cardiovascular Death, and Incident Chronic Kidney Disease: A National Health Insurance Service-National Sample Cohort in Korea
Source: Front Cardiovasc Med. 2022 May 2;9:882599. doi: 10.3389/fcvm.2022.882599 (PMC9108188; doi:10.3389/fcvm.2022.882599)

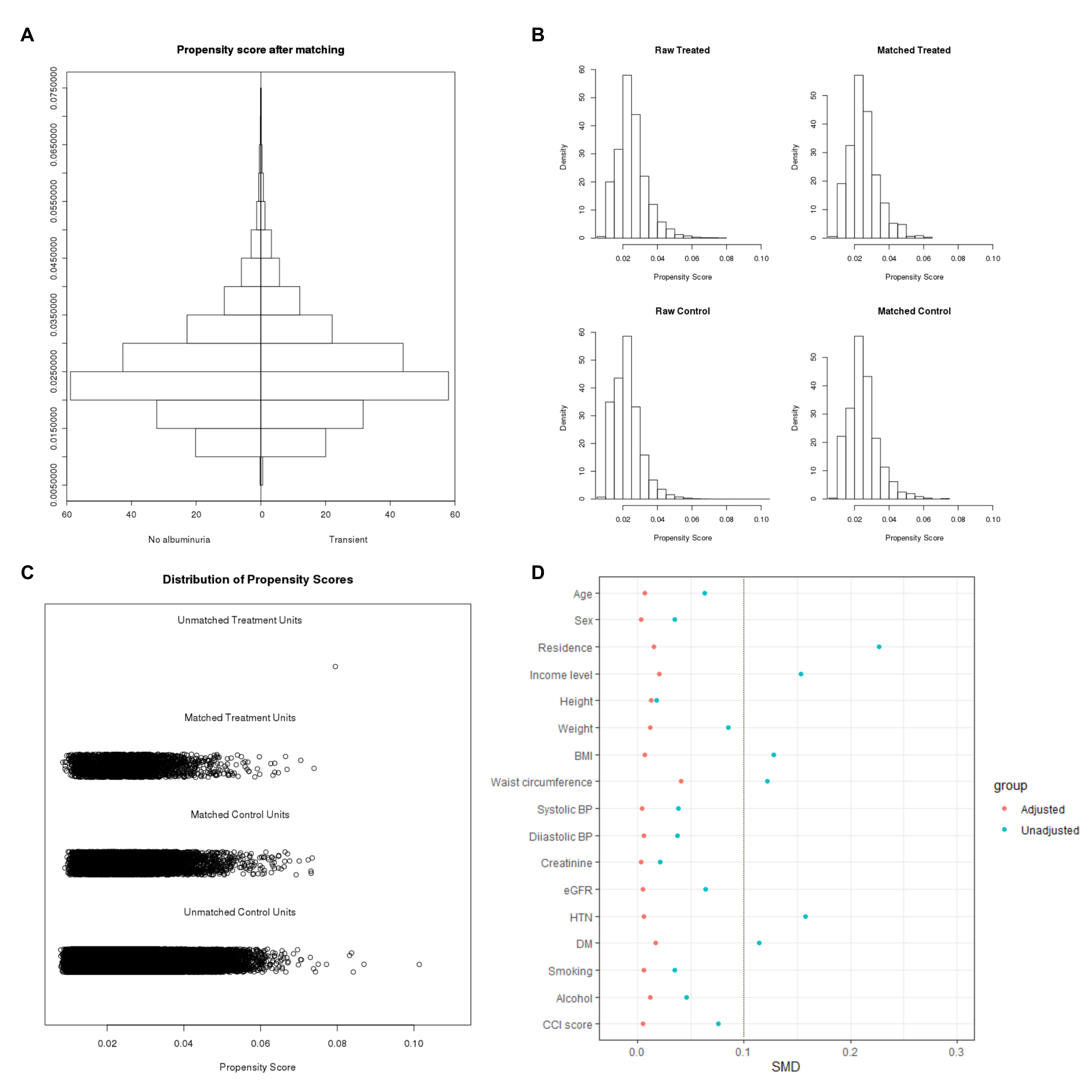

Supplement: Supplementary file 1 [file Image_1.TIF]

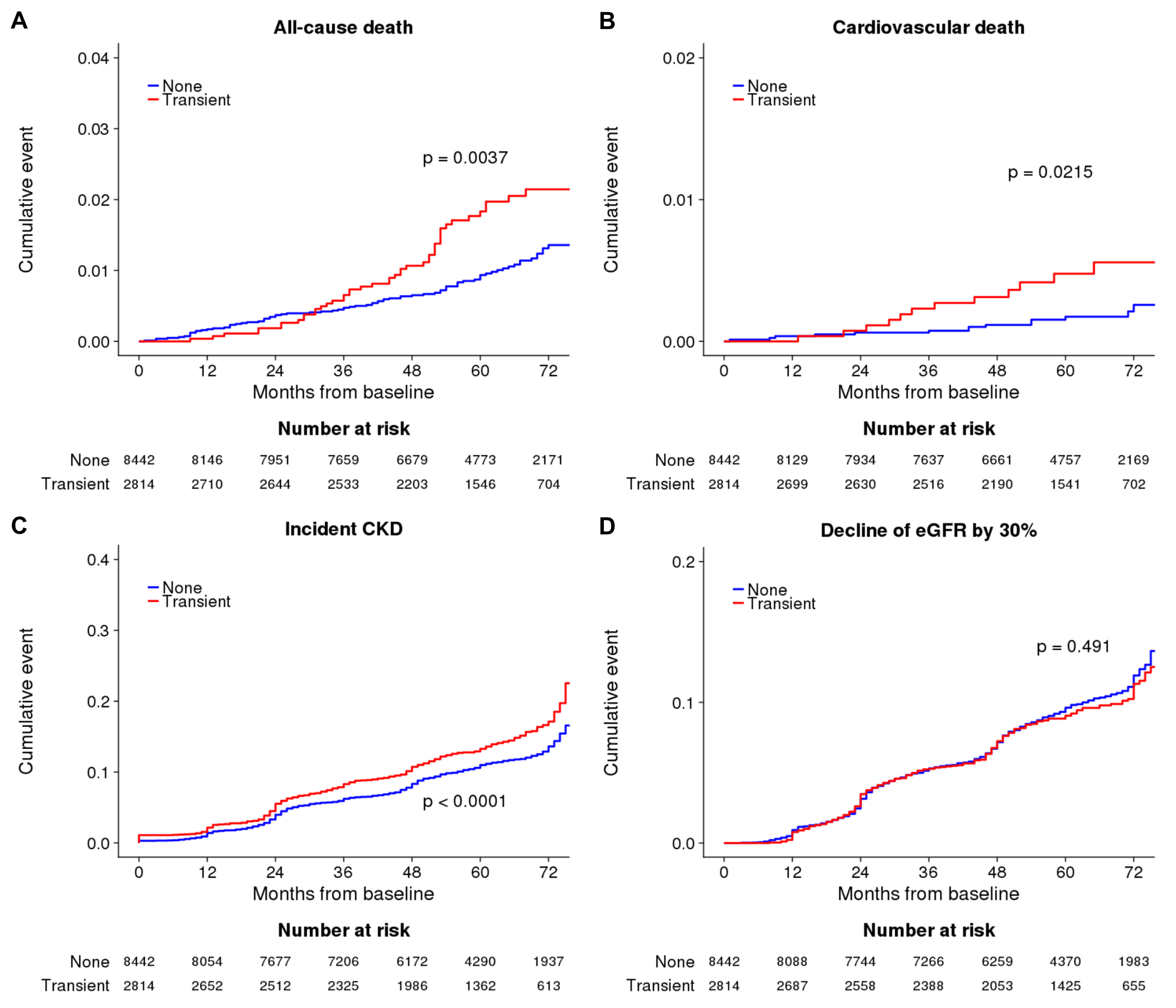

Supplement: Supplementary file 2 [file Image_2.TIF]
