# Supplementary material for: Transiently Observed Trace Albuminuria on Urine Dipstick Test Is Associated With All-Cause Death, Cardiovascular Death, and Incident Chronic Kidney Disease: A National Health Insurance Service-National Sample Cohort in Korea
Source: Front Cardiovasc Med. 2022 May 2;9:882599. doi: 10.3389/fcvm.2022.882599 (PMC9108188; doi:10.3389/fcvm.2022.882599)
Supplement: Supplementary file 3 [file Table_1.DOCX]

***Supplementary Material***

**1. Supplementary Methods**

***Study population***

Our study was based on the National Health Insurance Service-National Sample Cohort (NHIS-NSC) in Korea (1). The NHIS-NSC is composed of general health examination data of individuals from the general population in Korea. One can refer to relevant journals for more information (1, 2). In Korea, all individuals are covered by the NHIS and are required to participate.

The NHIS provides a unique general health examination program that measures anthropometry, including height, weight, waist circumference, and body mass index (BMI), blood pressure; laboratory tests, including lipid profile (total cholesterol, high-density lipoprotein [HDL]-cholesterol, and triglycerides), serum creatinine; and a urinalysis using the dipstick test. All adults aged 40 years or older, employees, and insured, self-employed householders are subject to a general health examination conducted biennially. Blue-collar employees receive general health examinations annually. This general health examination is compelled by law and is free of charge to all participants of the NHIS.

The NHIS established the National Health Information Database, which covers the entire population of Korea (50 million), and formed a representative cohort of 1,025,340 individuals (comprising 2.2% of the total population) from this database in 2002. The NHIS-NSC is exclusively serviced by the Korean government, which manages all medical services through the NHIS. Thus, the prescription lists, except over-the-counter drugs, and the International Classification of Diseases 10th revision (ICD-10) codes were included in this cohort. The cohort was followed up for 13 years, until 2015. If the participants were not censored by death, all medical data was available to the end of 2015. The rate of participation in the general health examination program for eligible individuals was 72.1%. As serum creatinine levels have been available since 2009 in the cohort, we targeted the population since 2009, including those aged >20 years.

***Structured data construction***

Figure 1 depicts the process of converting unstructured data into a structured form and including or excluding participants. Results of urine dipstick test showing negative (–), trace (±), positive (1+), and positive (≥2+) were considered to correspond to albumin-to-creatinine ratio levels of <10, 10–29, 30–299 (microalbuminuria), and ≥300 mg/g (macroalbuminuria), respectively (3). Although individual-level health examination programs are performed biennially, annual results are available for blue-collar employees. Therefore, we combined two consecutive years into one observation unit for the years 2005–2006, 2007–2008, and 2009–2010. When patients had negative urine dipstick test results (i.e., –/–/–) during three observational periods, they were assigned to the no albuminuria group. If they had a trace (±) dipstick test results in one of the first two observational periods and were negative in others (i.e., ±/–/– or –/±/–), they were assigned to the transient albuminuria group. If the urine dipstick test was negative in both the first two sequential observational periods but showed a trace result in the last observational period (i.e., –/–/±), they were excluded from the analysis because the results of the next follow-up urine dipstick tests were uncertain.

The third observation period (2009–2010) was selected as the baseline. Individuals registered as disabled at the baseline, those who died by the ICD-10 code of S or T (injury, poisoning, or other external causes) during the follow-up observation, or those who had a baseline serum creatinine level that was considered unreliable and imprecise (<0.4 mg/dL in men and <0.3 mg/dL in women) were excluded from the study. In addition, they might lead to overestimation of estimated glomerular filtration rates (eGFRs). Individuals >75 years of age and those with underlying CKD defined by their age-adapted eGFR threshold (75 ml/min per 1.73 m^2^ for those aged below 40 years; 60 ml/min per 1.73 m^2^ for those aged between 40 and 65 years; 45 ml/min per 1.73 m^2^ for those aged above 65 years) were also excluded (4). Although we attempted to exclude patients who had received renal replacement therapy due to ESKD by searching ICD-10 codes for maintenance dialysis (N185 and Z49) and kidney transplantation (T861 and Z941), no data were found to satisfy such conditions. People with missing data were excluded from the study.

***Covariates***

We set the years between 2009 and 2010, the last observation period, as a baseline. If health screening results for both years were available, we used the earlier one. Baseline characteristics, including age, sex, residence (urban vs. rural area), income level estimated based on health insurance expenditures (deciles), height (centimeters), weight (kg), BMI (kg/m^2^), waist circumference (cm), systolic blood pressure (BP, mmHg), diastolic BP (mmHg), serum creatinine levels, and eGFR calculated using the Chronic Kidney Disease Epidemiology Collaboration (CKD-EPI) equation (5) were collected.

For confounding factors, comorbidities were presented using the Charlson comorbidity index (CCI), which was calculated to represent and adjust comorbidities as an underlying burden from chronic diseases (6). Information concerning medical history, including hypertension (HTN) and diabetes mellitus (DM), was acquired using reports based on a self-reported questionnaire. When some details were unanswered or missing, ICD-10 codes were used to determine the medical history (I10–I15 for HTN and E10–E14 for DM). To calculate the CCI score, comorbidities were considered present when at least one or more inquiries for insurance existed at all years of the baseline period (2009–2010). To avoid missing cases with follow-up loss or cessation of medication without adequate reason (decisions against medical advice by self-estimation), we considered that there were comorbid diseases when there was at least one insurance request every year between 2006 and 2008. ICD-10 codes were searched in the same manner to determine the history of HTN and DM.

Lifestyle history of alcohol consumption and smoking was also acquired using reports based on a self-reported questionnaire. Alcohol consumption history was defined as none, moderate (up to two standard drinks per day for men and one standard drink per day for women), and heavy (more than a moderate amount) (7). Smoking history was defined as a current smoker or not (non- or ex-smoker). If there were missing values in the questionnaire for alcohol and smoking history, patients were excluded from further analysis.

Additionally, we attempted to adjust for non-HDL cholesterol, which was calculated by subtracting the HDL cholesterol from the total cholesterol. As this cohort was constructed by combining laboratory results from several clinics nationwide, it was considered that some outliers existed in the lipid profiles. Therefore, only those values with non-HDL cholesterol levels >0 were selected and analyzed separately. Additional results adjusted for non-HDL cholesterol as a covariate are presented separately.

***Study outcomes***

The outcomes of interest were as follows: (1) all-cause death, (2) cardiovascular death, (3) incident CKD, and (4) a decline in eGFR by more than 30%. All-cause death was defined as any death excluding those with an S or T code, according to the ICD-10 codes. Cardiovascular death was defined as death with a leading cause recorded as I code by the ICD-10 (I00 to I99). Incident CKD was defined as either eGFR or albuminuria. The age-adapted eGFR threshold was used for eGFR criteria (4). Albuminuria was defined as a urine dipstick test result of 1+ or greater based on previous studies (8, 9). A decline in eGFR by more than 30% was also based on previous studies, although the strength of evidence was slightly weaker than that of doubling of serum creatinine or a decline in GFR by more than 40% (10).

***Statistical analysis***

All statistical analyses were performed using R version 3.3 (The R Foundation for Statistical Computing, Vienna, Austria). Categorical variables are expressed as counts and percentages. Continuous variables are expressed as mean ± standard deviation (SD). The differences between groups were compared using the Student’s t-test or Mann-Whitney test, as appropriate.

The Cox proportional hazard model was used to evaluate the associations between transient albuminuria and the risk of defined outcomes. The no albuminuria group was used as a reference for comparison with the transient albuminuria group. The multivariable model adjusted for confounding factors, including age, sex, residence, income level, BMI, systolic BP, eGFR, CCI score, history of HTN and DM, and smoking and alcohol status. The proportional hazard assumption was estimated using Schoenfeld residuals. We tested Schoenfeld residuals by stratifying categorical variables one by one until all covariates had a p-value >0.05. Continuous variables were transformed into categorical variables if no categorical variables had *p*<0.05 anymore and some continuous variables had *p*>0.05. Information can be lost when continuous variables are transformed into categorical variables; thus, conversions were only performed when the *p*-values of all other categorical variables were higher than 0.05, and those of continuous variables were less than 0.05. When converting a continuous variable into a categorical type, age was categorized as <40, 40–59, and >60 years; eGFR was categorized as 45–59, 60–74, 75–89, 90–105, and ≥105 ml/min per 1.73 m^2^; BMI was categorized as <18.5, 18.5–24.9, 25–29.9, and ≥30; and CCI scores were categorized as 1, 2, 3, 4, 5, and ≥6. Stratification was performed until the *p*-values of all covariates were higher than 0.05.

We used 1:3 propensity matching and the nearest method with a caliper of 0.25, based on the method described by Rosenbaum and Rubin, to remove at least 90% of bias (11). A logistic regression model was used to estimate the propensity score between the transient albuminuria and no albuminuria groups (12, 13). The propensity score was regressed with baseline characteristics considered as covariates to be adjusted for in the Cox proportional hazard model because covariates that affected outcomes are better for estimating the propensity score to regress the treatment group (14). Thus, age, sex, residence, income level, BMI, systolic BP, eGFR, CCI score, history of HTN and DM, and smoking and alcohol status were used as covariates. Matching between the two groups was based on propensity scores using the “MatchIt” package of R software. After propensity scores were estimated, quantiles of the estimated propensity scores were computed to be stratified into quartiles (12). Survival analysis was performed using the stratified log-rank test based on propensity scores (15). To quantify differences between the two groups, standardized differences were calculated because statistical significance tests could not be used to compare baseline characteristics between propensity score-matched data (12). The threshold for the standardized difference was set at 0.1. Two-sided *p*-values <0.05 were considered to be statistically significant.

***Sensitivity analysis***

When the difference in the number of participants between the two groups was large, there was a concern that the result might vary depending on which individuals were selected during the matching process. To address this problem, a sensitivity analysis using bootstrapping was performed. Our simulation process was adopted from a previous study (16). One thousand subsamples were randomly obtained from the no albuminuria group without replacement. Each subsample extracted 10% of the individuals from the no albuminuria group. These subsampling processes were conducted to make the ratio of the study outcome (all-cause death and cardiovascular death) constant. Next, the transient albuminuria group was added to the subsamples. Propensity matching between the transient albuminuria group and the subsampled no albuminuria group was then performed. As a result, 1,000 propensity score-matched subgroups were created. These matching processes were performed using the nearest method, a caliper of 0.25, and a ratio of 1:1. Propensity scores were estimated using variables that were considered as confounders to be adjusted for in the Cox proportional hazard model as described above, including age, sex, residence, income level, BMI, systolic BP, eGFR, CCI score, history of HTN, DM, smoking, and alcohol history. The hazard ratio was adjusted by propensity score strata using the Cox regression model (12, 15). The mean hazard ratio and 95% confidence intervals (CIs) were obtained by nonparametric percentile-based estimates (i.e., 2.5th and 97.5th percentiles) through a combination of propensity score matching and bootstrapping.

**References**

(1) Lee J, Lee JS, Park SH, Shin SA, Kim K. Cohort Profile: The National Health Insurance Service-National Sample Cohort (NHIS-NSC), South Korea. Int J Epidemiol. 2017 Apr 1;46(2):e15. Epub 2016/01/30. doi:10.1093/ije/dyv319. Cited in: Pubmed; PMID 26822938.

(2) Kim KM, Oh HJ, Choi HY, Lee H, Ryu DR. Impact of chronic kidney disease on mortality: A nationwide cohort study. Kidney Res Clin Pract. 2019 Sep 30;38(3):382-390. Epub 2019/08/07. doi:10.23876/j.krcp.18.0128. Cited in: Pubmed; PMID 31382730.

(3) Hallan SI, Matsushita K, Sang Y, Mahmoodi BK, Black C, Ishani A, Kleefstra N, Naimark D, Roderick P, Tonelli M, Wetzels JF, Astor BC, Gansevoort RT, Levin A, Wen CP, Coresh J, Chronic Kidney Disease Prognosis C. Age and association of kidney measures with mortality and end-stage renal disease. JAMA. 2012 Dec 12;308(22):2349-60. Epub 2012/11/01. doi:10.1001/jama.2012.16817. Cited in: Pubmed; PMID 23111824.

(4) Delanaye P, Jager KJ, Bokenkamp A, Christensson A, Dubourg L, Eriksen BO, Gaillard F, Gambaro G, van der Giet M, Glassock RJ, Indridason OS, van Londen M, Mariat C, Melsom T, Moranne O, Nordin G, Palsson R, Pottel H, Rule AD, Schaeffner E, Taal MW, White C, Grubb A, van den Brand J. CKD: A Call for an Age-Adapted Definition. J Am Soc Nephrol. 2019 Oct;30(10):1785-1805. Epub 2019/09/12. doi:10.1681/ASN.2019030238. Cited in: Pubmed; PMID 31506289.

(5) Inker LA, Schmid CH, Tighiouart H, Eckfeldt JH, Feldman HI, Greene T, Kusek JW, Manzi J, Van Lente F, Zhang YL, Coresh J, Levey AS, Investigators C-E. Estimating glomerular filtration rate from serum creatinine and cystatin C. N Engl J Med. 2012 Jul 5;367(1):20-9. Epub 2012/07/06. doi:10.1056/NEJMoa1114248. Cited in: Pubmed; PMID 22762315.

(6) Sundararajan V, Henderson T, Perry C, Muggivan A, Quan H, Ghali WA. New ICD-10 version of the Charlson comorbidity index predicted in-hospital mortality. J Clin Epidemiol. 2004 Dec;57(12):1288-94. Epub 2004/12/25. doi:10.1016/j.jclinepi.2004.03.012. Cited in: Pubmed; PMID 15617955.

(7) DeSalvo KB, Olson R, Casavale KO. Dietary Guidelines for Americans. JAMA. 2016 Feb 2;315(5):457-8. Epub 2016/01/10. doi:10.1001/jama.2015.18396. Cited in: Pubmed; PMID 26746707.

(8) White SL, Yu R, Craig JC, Polkinghorne KR, Atkins RC, Chadban SJ. Diagnostic accuracy of urine dipsticks for detection of albuminuria in the general community. Am J Kidney Dis. 2011 Jul;58(1):19-28. Epub 2011/03/18. doi:10.1053/j.ajkd.2010.12.026. Cited in: Pubmed; PMID 21411199.

(9) Sumida K, Nadkarni GN, Grams ME, Sang Y, Ballew SH, Coresh J, Matsushita K, Surapaneni A, Brunskill N, Chadban SJ, Chang AR, Cirillo M, Daratha KB, Gansevoort RT, Garg AX, Iacoviello L, Kayama T, Konta T, Kovesdy CP, Lash J, Lee BJ, Major RW, Metzger M, Miura K, Naimark DMJ, Nelson RG, Sawhney S, Stempniewicz N, Tang M, Townsend RR, Traynor JP, Valdivielso JM, Wetzels J, Polkinghorne KR, Heerspink HJL, Chronic Kidney Disease Prognosis C. Conversion of Urine Protein-Creatinine Ratio or Urine Dipstick Protein to Urine Albumin-Creatinine Ratio for Use in Chronic Kidney Disease Screening and Prognosis : An Individual Participant-Based Meta-analysis. Ann Intern Med. 2020 Sep 15;173(6):426-435. Epub 2020/07/14. doi:10.7326/M20-0529. Cited in: Pubmed; PMID 32658569.

(10) Levey AS, Gansevoort RT, Coresh J, Inker LA, Heerspink HL, Grams ME, Greene T, Tighiouart H, Matsushita K, Ballew SH, Sang Y, Vonesh E, Ying J, Manley T, de Zeeuw D, Eckardt KU, Levin A, Perkovic V, Zhang L, Willis K. Change in Albuminuria and GFR as End Points for Clinical Trials in Early Stages of CKD: A Scientific Workshop Sponsored by the National Kidney Foundation in Collaboration With the US Food and Drug Administration and European Medicines Agency. Am J Kidney Dis. 2020 Jan;75(1):84-104. Epub 2019/09/02. doi:10.1053/j.ajkd.2019.06.009. Cited in: Pubmed; PMID 31473020.

(11) Rosenbaum PR, Rubin DB. Constructing a Control Group Using Multivariate Matched Sampling Methods That Incorporate the Propensity Score. The American Statistician. 1985;39(1):33-38. Full publication date: Feb., 1985. doi:10.2307/2683903.

(12) Austin PC. A Tutorial and Case Study in Propensity Score Analysis: An Application to Estimating the Effect of In-Hospital Smoking Cessation Counseling on Mortality. Multivariate Behav Res. 2011;46(1):119-151. Epub 2012/01/31. doi:10.1080/00273171.2011.540480. Cited in: Pubmed; PMID 22287812.

(13) Austin PC. An Introduction to Propensity Score Methods for Reducing the Effects of Confounding in Observational Studies. Multivariate Behav Res. 2011 May;46(3):399-424. Epub 2011/08/06. doi:10.1080/00273171.2011.568786. Cited in: Pubmed; PMID 21818162.

(14) Austin PC, Grootendorst P, Anderson GM. A comparison of the ability of different propensity score models to balance measured variables between treated and untreated subjects: a Monte Carlo study. Stat Med. 2007 Feb 20;26(4):734-53. Epub 2006/05/19. doi:10.1002/sim.2580. Cited in: Pubmed; PMID 16708349.

(15) Austin PC. The use of propensity score methods with survival or time-to-event outcomes: reporting measures of effect similar to those used in randomized experiments. Stat Med. 2014 Mar 30;33(7):1242-58. Epub 2013/10/15. doi:10.1002/sim.5984. Cited in: Pubmed; PMID 24122911.

(16) Austin PC, Small DS. The use of bootstrapping when using propensity-score matching without replacement: a simulation study. Stat Med. 2014 Oct 30;33(24):4306-19. Epub 2014/08/05. doi:10.1002/sim.6276. Cited in: Pubmed; PMID 25087884.

**2. Supplementary Tables**

Supplementary Table 1. Charlson comorbidity index score and concomitant comorbidities in both groups.

| Variables | No albuminuria  (N = 121,876) | Transient albuminuria  (N = 2,815) |
| --- | --- | --- |
| The Charlson comorbidity index score | 0.4 ± 0.8 | 0.4 ± 0.9 |
| 0 score | 91327 (74.9) | 2061 (73.3) |
| 1 score | 20847 (17.1) | 457 (16.1) |
| 2 score | 6350 (5.2) | 182 (6.5) |
| 3 score | 2165 (1.8) | 67 (2.4) |
| 4 score | 748 (0.6) | 28 (1.0) |
| 5 score | 302 (0.2) | 12 (0.4) |
| ≥6 score | 137 (0.1) | 5 (0.2) |
| Myocardial infarction, n (%) | 266 (0.2) | 10 (0.4) |
| Congestive heart failure, n (%) | 323 (0.3) | 11 (0.4) |
| Peripheral vascular disease, n (%) | 2686 (2.2) | 74 (2.6) |
| Cerebrovascular disease, n (%) | 2585 (2.1) | 78 (2.8) |
| Dementia, n (%) | 0 (0.0) | 0 (0.0) |
| Chronic pulmonary disease, n (%) | 11138 (9.1) | 266 (9.4) |
| Rheumatologic disease, n (%) | 815 (0.7) | 19 (0.7) |
| Peptic ulcer disease, n (%) | 12983 (10.7) | 283 (10.1) |
| Mild liver disease, n (%) | 1204 (1.0) | 37 (1.3) |
| Diabetes without chronic complications, n (%) | 6284 (5.2) | 218 (7.7) |
| Diabetes with chronic complications, n (%) | 1304 (1.1) | 59 (2.1) |
| Hemiplegia or paraplegia, n (%) | 40 (0.0) | 3 (0.1) |
| Renal disease, n (%) | 137 (0.1) | 4 (0.1) |
| Any malignancy, n (%) | 1734 (1.4) | 42 (1.5) |
| Metastatic solid tumor, n (%) | 126 (0.1) | 3 (0.1) |
| Moderate or severe liver disease, n (%) | 109 (0.1) | 3 (0.1) |
| AIDS/HIV, n (%) | 0 (0.0) | 0 (0.0) |

Abbreviations: AIDS, acquired immunodeficiency syndrome; HIV, human immunodeficiency virus.

Supplementary Table 2. Income level was estimated based on the health insurance expenditure in both the groups.

| Income level, n (%) | No albuminuria  (N = 121,876) | Transient albuminuria  (N = 2,815) |
| --- | --- | --- |
| Decile 1, 6938 (5.6) | 6803 (5.6) | 135 (4.8) |
| Decile 2, 6391 (5.1) | 6233 (5.1) | 158 (5.6) |
| Decile 3, 7208 (5.8) | 7068 (5.8) | 140 (5.0) |
| Decile 4, 8005 (6.4) | 7846 (6.4) | 159 (5.6) |
| Decile 5, 10918 (8.8) | 10707 (8.8) | 211 (7.5) |
| Decile 6, 12825 (10.3) | 12564 (10.3) | 261 (9.3) |
| Decile 7, 15532 (12.5) | 15213 (12.5) | 319 (11.3) |
| Decile 8, 18338 (14.7) | 17943 (14.7) | 395 (14.0) |
| Decile 9, 20771 (16.7) | 20267 (16.6) | 504 (17.9) |
| Decile 10, 17765 (14.2) | 17232 (14.1) | 533 (18.9) |

Decile 10 had the highest income, while decile 1 was the poorest group.

Supplementary Table 3. Relative hazard ratio of outcomes in the transient albuminuria group compared to the no albuminuria group, after additional adjustment using non-HDL cholesterol.

| Outcome | Adjusted hazard ratio | *p*-value |
| --- | --- | --- |
| All-cause mortality^*^ |  |  |
| None | Reference |  |
| Transient | 1.34 (0.96 – 1.88) | 0.087 |
| Cardiovascular mortality^†^ |  |  |
| None | Reference |  |
| Transient | 1.76 (1.00 – 3.07) | 0.048 |
| Incident CKD^‡^ |  |  |
| No albuminuria | Reference |  |
| Transient | 1.29 (1.16 – 1.43) | < 0.001 |
| > 30% decline in eGFR^§^ |  |  |
| No albuminuria | Reference |  |
| Transient | 1.02 (0.86 – 1.12) | 0.755 |

124,519 individuals remained after exclusion of 172 individuals whose non-HDL cholesterol level was ≤0.

Age, sex, residence, income level, BMI, systolic BP, baseline eGFR, Charlson comorbidity index score, history of HTN and DM, smoking and alcohol status, and non-HDL cholesterol levels were included in the model to adjust for other confounders. Whether confounders would be used as covariates or strata was determined based on Schoenfeld residuals.

^*^ BMI, systolic BP, baseline eGFR, and non-HDL cholesterol levels were used as covariates, while the others were used as strata.

^†^ Residence, BMI, systolic BP, eGFR, smoking and alcohol status, history of HTN and DM, Charlson comorbidity index score, and non-HDL cholesterol levels were used as covariates, and others were used as strata.

^‡^ Residence, BMI, systolic BP, baseline eGFR, smoking and alcohol status, history of DM, and non-HDL cholesterol levels were used as covariates and others were used as strata.

^§^ Residence, BMI, systolic BP, history of DM, Charlson comorbidity index score, and non-HDL cholesterol levels were used as covariates, and others were used as strata.

Abbreviations: HDL, high-density lipoprotein; CKD, chronic kidney disease; eGFR, estimated glomerular filtration rate; BMI, body mass index; BP, blood pressure; HTN, hypertension; DM, diabetes mellitus.

**3. Supplementary Figures legend**

**Supplementary Figure 1. Plot to show balance diagnostics for propensity score matching.** (A) Back-to-back histogram after matching, (B) histograms displaying the density of propensity scores before and after matching, (C) distribution of propensity scores, and (D) covariate balance measured using standardized difference before and after matching.

**Supplementary Figure 2. Kaplan-Meier curves of adverse outcomes after propensity score matching for transient albuminuria and no albuminuria groups.** The red line denotes the transient albuminuria group; the blue line indicates the no albuminuria group. Adverse outcomes included all-cause death (A), cardiovascular death (B), incident CKD (C), and a decline in eGFR by more than 30% from the baseline (D).

***Abbreviations:*** CKD, chronic kidney disease; eGFR, estimated glomerular filtration rate.
